# Steel Rod Magnetised by the Influence of the Will

**Published:** 1844-12

**Authors:** 


*Magnetization of a Steel Rod by the mere Influence of the Will!*—M. Thilorier, on the 11th and 18th of June last, endeavored to establish, by a series of experiments, before the French Academy of Sciences, the existence in the human body of a fluid or agency analogous to the electric, and capable of producing some very curious phenomena. Many facts, he said, might be adduced to prove this position, which will probably startle most who have not thought of the subject. Of these, one of the most satisfactory is that of magnetizing a steel rod, placed upon the epigastrium, by the mere and unaided effort of the will! By the simple act of volition, this effect is produced; for it is well known that the mere contact of the rod with the surface of the body is not sufficient. M. Thilorier says that there are three principal points of the body from which the magnetic fluid escapes, viz., the hands, the epigastrium, and the forehead! In a state of repose or passiveness of the intellect and will, as for example during sleep, the efluvium, and consequently the magnetization, are not however entirely null. In the hands, the currents of the fluid are parallel to the direction of the fingers, and perpendicular to the palm; those of the epigastric and frontal regions are directed from below upwards, and from the feet to the head, in a curved line! The magnetic action is null, or nearly so, at the lower end of the spinal marrow, it increases in degree or intensity in proportion as we approach the neck! Currents emanate from every point of the cranial vault; but the most energetic of all is from the culminating point of the frontal bone! The energy of the currents depends on a variety of circumstances; it is more considerable about noon than at night, or in the morning; likewise more so in health than in sickness!

*Med. Chir. Review, from L'Expèrience.*

